# Laser Welding and Syncristallization Techniques Comparison: *In Vitro* Study

**DOI:** 10.1155/2012/720538

**Published:** 2012-06-21

**Authors:** C. Fornaini, E. Merigo, P. Vescovi, M. Meleti, S. Nammour

**Affiliations:** ^1^Department of Dental Sciences, Faculty of Medicine, University of Liège, 4000 Liège, Belgium; ^2^Oral Medicine and Laser-Assisted Surgery Unit, Dental School, Faculty of Medicine and Surgery, University of Parma, 12-143121 Parma, Italy

## Abstract

*Background*. Laser welding was first reported in 1967 and for many years it has been used in dental laboratories with several advantages versus the conventional technique. Authors described, in previous works, the possibility of using also chair-side Nd : YAG laser device (Fotona Fidelis III, *λ* = 1064 nm) for welding metallic parts of prosthetic appliances directly in the dental office, extra- and also intra-orally. Syncristallisation is a soldering technique based on the creation of an electric arc between two electrodes and used to connect implants to bars intra-orally. *Aim*. The aim of this study was to compare two different laser welding devices with a soldering machine, all of these used in prosthetic dentistry. *Material and Methods*. In-lab Nd : YAG laser welding (group A = 12 samples), chair-side Nd : YAG laser welding (group B = 12 samples), and electrowelder (group C = 12 samples) were used. The tests were performed on 36 CrCoMo plates and the analysis consisted in evaluation, by microscopic observation, of the number of fissures in welded areas of groups A and B and in measurement of the welding strength in all the groups. The results were statistically analysed by means of one-way ANOVA and Tukey-Kramer multiple comparison tests. *Results*. The means and standard deviations for the number of fissures in welded areas were 8.12 ± 2.59 for group A and 5.20 ± 1.38 for group B. The difference was statistical significant (*P* = 0.0023 at the level 95%). On the other hand, the means and standard deviations for the traction tests were 1185.50 ± 288.56 N for group A, 896.41 ± 120.84 N for group B, and 283.58 ± 84.98 N for group C. The difference was statistical significant (*P* = 0.01 at the level 95%). *Conclusion*. The joint obtained by welding devices had a significant higher strength compared with that obtained by the electrowelder, and the comparison between the two laser devices used demonstrated that the chair-side Nd : YAG, even giving a lower strength to the joints, produced the lowest number of fissures in the welded area.

## 1. Introduction

In 1967, Gordon described the possibility of welding the metallic portions of dental prosthesis using a laser and this technique has been used since the 1970s in dental laboratories, rapidly demonstrating its advantages over traditional welding methods [1]. 

In fact, the procedure can be carried out directly on the master cast, thereby eliminating the risk of inaccuracies and distortions due to the duplication of the model [2]. Moreover, the heat source is a concentrated high-power light beam, so minimizing distortion problems in the prosthetic pieces [3]. The process allows the possibility of welding adjacent to acrylic resin or ceramic parts with neither physical (cracking) nor colour damage [4], thereby allowing a reduction of working time by eliminating the necessity to remake broken prosthetic or orthodontic appliances. 

Laboratory tests have shown that laser-welded joints have a high, reproducible strength [5]. Laser welding technique has been used for many years in dental technician laboratory to manufacture prosthetics by connecting the different pieces and in repairing broken appliances. Unfortunately, there are more very important disadvantages such as costs and sizes of devices, and also the difficulty in the management of the parameters, which needs a long training period and makes this technique strictly operator dependent.

In previous works, authors have described the possibility of using the same Nd : YAG laser used in dental office for surgery interventions to weld metallic pieces of prosthetic and orthodontic appliances and due to fiberoptic delivery system of this device, they also proposed the possibility of direct intra-oral welding by dentists themselves [6].

A further different method, described for intra-oral welding, is based on the creation of an electric arc between two electrodes under an argon gas flux and it is called “syncristallisation” [7, 8]; unfortunately, there are more limits: it is not effective on every kind of metal and alloy, and it cannot be used on patients with pacemakers, it cannot work with filler metal, and some of the energy necessary for the welding process, which is concentrated between the two electrodes, spreads to the adjacent area (teeth, acrylic, ceramic, etc.).

 The laser welding technique, as described before, is effective on all metals and alloys, can be applied either with or without filler metal and shielding gas, and, due to the extremely small spot size of the beam (0.6 mm), is able to limit the high temperature required to a very limited area. 

Furthermore, it can be used on all patients and does not require a new and specific appliance, but utilizes an appliance currently available for oral treatments in the dental office.

The aim of this study is to compare the welding process obtained by three different devices: an “in-lab Nd : YAG laser welding,” a “chair-side Nd : YAG laser welding,” and a “Syncristallisation machine,” by analysing the strength of the joints and by microscopic observation of the samples, in order to determine the more proper technique for clinical use.

## 2. Materials and Methods

**Figure 1:**
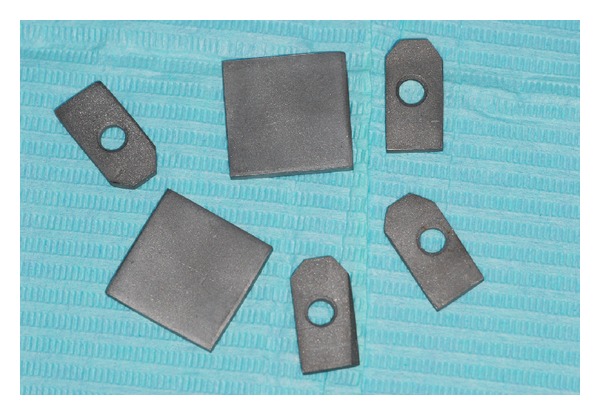
The metal plates used in the tests.

Thirty-six plates of 20 × 20 × 1 mm dimension were divided into three groups of twelve samples (1A, 1B, and 1C), and thirty-six CoCrMo plates of 8 × 29 × 1 mm dimension were divided into three groups of twelve samples (2A, 2B, and 2C); on each plate of group 2 a hole of 3 mm diameter was performed (Figure 1). 

**Figure 2:**
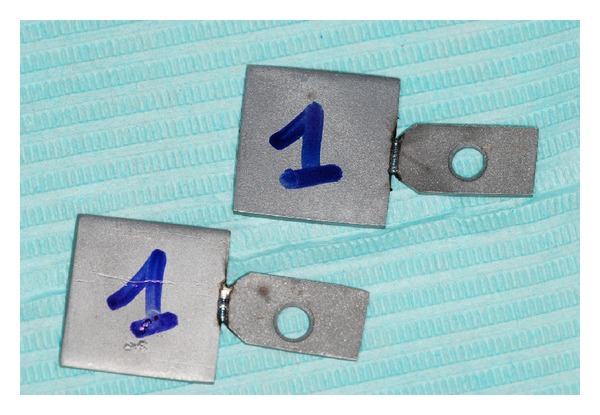
Plates of group A welded by in-lab Nd : YAG laser welding.

Each plate of group 1A was welded to a plate of group 2A by In-lab Nd : YAG laser welding; the two parts were edge-to-edge connected (Figure 2). 

The device used was Titec LASER 50 L (Orotig, Brescia, Italy) with these parameters: Wavelength 1064 nm, beam spot 1.8 mm, peak power 4.3 kW, working distance: 15 mm. Volt 270, energy/pulse 2.7 J, 4.0 Hz frequency, pulse duration 2.3 msec, output power 2.4 KW, fluence 1516 J/cm^2^.

**Figure 3:**
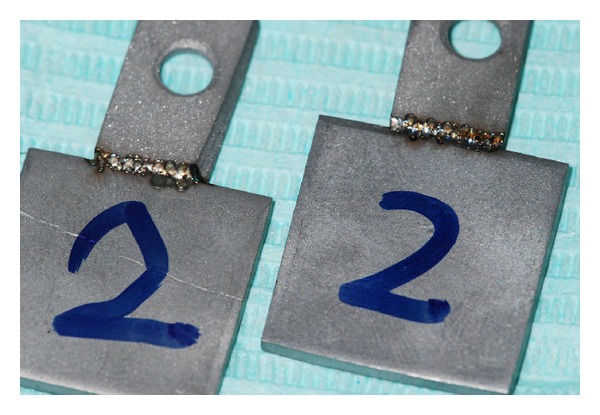
Plates of group B welded by chair-side Nd : YAG laser welding.

A single passage without metal filler was performed. Each plate of group 2A was welded to a plate of group 2B by chair-side Nd : YAG laser welding, the two parties were edge-to-edge connected (Figure 3).

The device used was Fidelis III Plus (Fotona, Ljubljana, Slovenia), with these parameters: Wavelength 1064 nm, output power 9.85 W, frequency 1 Hz, pulse duration 15 msec, spot diam 0.6 mm, working distance 40 mm, energy 9. 85 J, fluence 3300 J/cm^2^.

Due to the optic fiber delivery system (900 *μ*m diameter), a power meter was used to check if there was no loss of energy (Ophir Nova II, thermal head F150A, Israel).

A single passage without metal filler was performed. The parameters used were the “standard” for each device and the fluence values were very different, due to the smallest laser beam diameter and longer pulse duration of the chair-side Nd : YAG laser welding.

**Figure 4:**
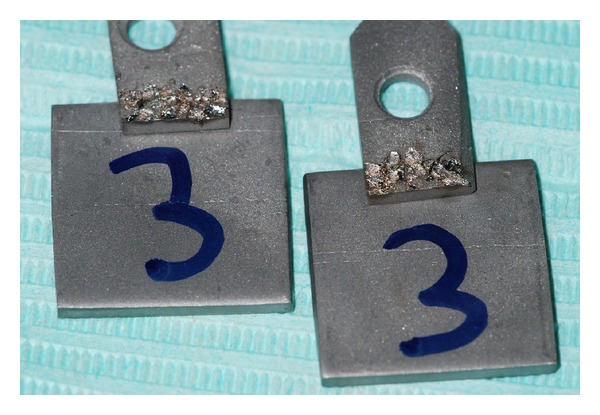
Plates of group C soldered by the electrowelder.

Each plate of group 3A was soldered with a plate of group 3B by an electrowelder (Figure 4) using the syncristallisation; in this case, due to the limit of this technique (creation of an electrical current through an electrode), it was not possible to connect the two parts in an edge-to-edge mode, so they were soldered one over the other with an overlapping of 3 mm.

The device used was VISION STRATEGICA (Newmed, Reggio Emilia, Italy) with these parameters: 25 V, 50 Hz, and 312 J.

The soldering process was done by a series of points because with this technique it is not possible to use metal filler.

Twenty-four plates, twelve of group A and twelve of group B, were observed by two different operators with optical microscope (Novex zoom Stereo RZ, Euromex Microscopes, Netherland) in order to count the number of fissures present in each plate. The values were statistically analysed with Students *t*-test. 

Then all the thirty-six welded plates, (twelve of the group A, twelve of the group B, and twelve of the group C), were connected to a dynamometer system (SBS-KW-300A, Steinberg, Berlin, GER) by means of a bar inserted in the hole.

 Each plate was clamped, on the opposite side, in a wood vice mounted on the base of the stand. Then traction was applied until the two parts were broken. All the values of the traction tests of all groups were reported and statistically analysed using one-way (ANOVA) and Tukey-Kramer Multiple comparison test.

## 3. Results

**Figure 5:**
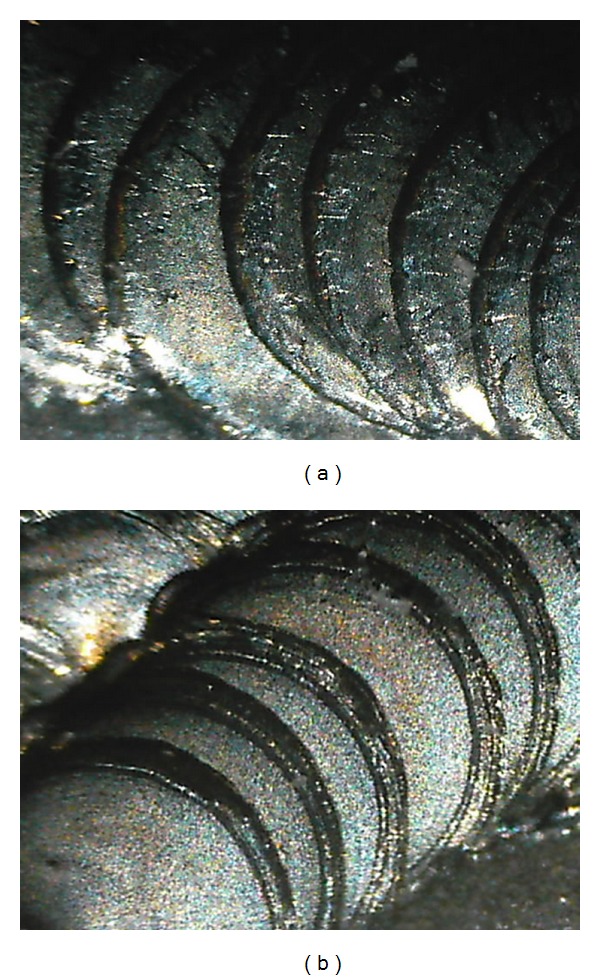
Microscopic vision of group A (a) and group B (b) laser welded plates.

The microscopic observation was limited to the plates of groups A and B in order to compare the number of fissures present (Figure 5).

In group A the highest score was 12 and the minimum was 4, the mean and standard deviation were 8.12 ± 2.59. 

**Figure 6:**
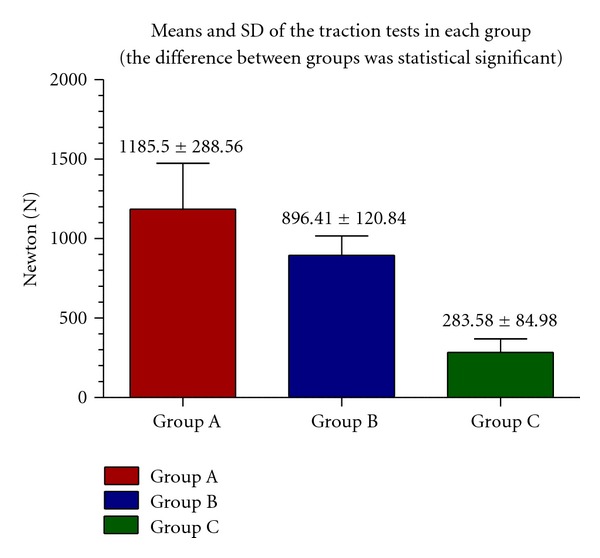
Mean and SD of the number of fissures in groups A and B.

In group B the highest score was 8 and the minimum was 3, the mean and standard deviation were 5.2 ± 1.38 (Figure 6).

The *T* Student test showed that the difference between the means was significant (*P* = 0.0023 at the level 95%).

On the other hand, the traction tests on group A pointed out that the highest value (expressed in N) was 1708 and the minimum was 870, the mean and the standard deviation were 1185.5 ± 288.56 N.

In the traction tests on group B, the highest value (expressed in N) was 1077 and the minimum was 670, the mean and the standard deviation were 896.41 ± 120.84 N.

In the traction test on group C, the highest value (expressed in N) was 402 and the minimum was 172, the mean and the standard deviation were 283.58 ± 84.98 N.

**Figure 7:**
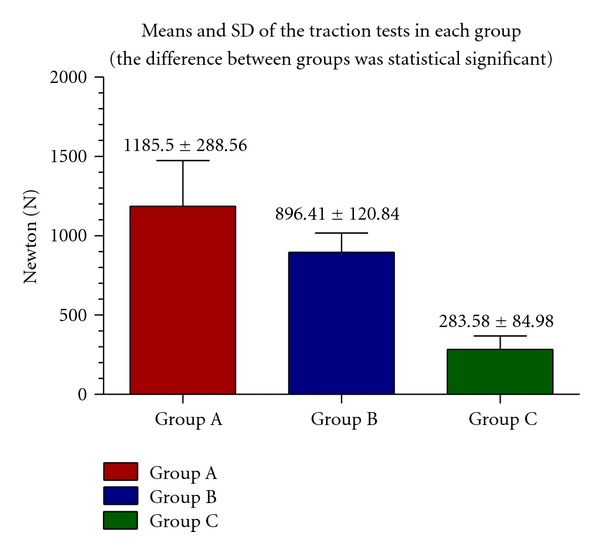
Mean and SD of traction tests in each group.

The mean values and SD of traction tests in each group are reported in Figure 7.

ANOVA statistical tests showed that the difference between the means of traction tests were significant (*P* = 0.01 at the level 95%). 

## 4. Discussion

While the comparison between the two laser devices was not difficult due to the similar welding process, the comparison between the results of laser welding and electrowelding was not easy, due to the great differences in procedures between the techniques.

However, because to date they are the only two ways to make an intra-oral welding, the effort to compare them, even with a lot of difficulties, was justified.

Laser technology is the most efficient method for applying thermal energy to small areas and, according to many Authors [9, 10], it is one of the best fusion-welding techniques for dissimilar metals. This depends on the possibility of modern laser appliances to focus the light beam on a very reduced focal point. This beam imparts energy into the metal causing it to heat up locally to a temperature above the liquid. So, the metal evaporates, a cavity is formed immediately under the heat source and a reservoir of melted metal is produced around it. As the heat source moves forward, the hole is filled with the melted metal from the reservoir and this solidifies to form the weld bead [11]. The best advantage is that the weld can usually be placed exactly where it is required, that is, at the point of workpiece abutment [12].

Hot cracking susceptibility during welding is usually evaluated when the strain or stress is changed during the process, but the use of a pulsed Nd : YAG laser, where power is continuously decreased with time, may control the rate of solidification and can effectively reduce hot cracking in alloys [13]. The cracks are generated during or after welding, and they are determined by the laser output, spot diameter, and laser beam diameter, [14]. This might explain the difference in the fissures numbers observed between group A and B samples.

Authors, in previous works, demonstrated by *in vitro* study on bovine jaws that during the welding process by Chair-side Nd : YAG laser welding, the temperature in the surrounding structures, in particular the pulp chamber, is very low and biologically harmless [15].

The so-called “syncristallisaton” technique was introduced in dentistry at the end of the 70s [16]. Despite the fact that many clinical cases are described in several works [17], there is a lack of *in vitro* studies on the physical mechanisms and the thermal elevations in the biological tissues. This makes it still very difficult to do a review of the literature.

The process of syncristallisation consists in an atoms movement resulting in the creation of a crystalloid structure in the area of junction [18]. The solder exploits the high temperature generated on the welding surface for a time of two thousands of a second and less. This is due to the resistance of the metals to the electric current flow and works by binding all those materials, such as titanium, surgical steel and nonnoble metal alloys, which are poor conductors of electricity [19].

Thanks to the very low conductibility of these metals and alloys and to the brevity of the exposure to the electric current, no tissue damage seems to result from this procedure [20] even if *in vitro* studies on the thermal elevation are very few.

Furthermore, unlike industrial solders that can operate only in the presence of argon and without oxygen in the atmosphere, the electrowelder used in dentistry works in the presence of oxygen, water, physiological oral fluids, and blood [21].

In this study the electrowelder seemed to give the lowest joint traction test values compared to the laser welding techniques, even if it is simpler, faster, and without parameters to adjust. Moreover, the technique of the syncristallisation has a great limitation consisting in the possibility to weld only with an overlapping of the two portions.

The best values in the mechanical tests were given by the plates welded by in-lab Nd: YAG laser welding even if, probably due to the higher energy delivered, the number of fissures noticed was higher than that observed in the plates welded by a chair-side Nd: YAG laser welding. Next studies about the application of these welding techniques will regard the thermal elevation comparison by *ex vivo* tests on implants in bovine jaws.

## 5. Conclusion

The use of the chair-side Nd : YAG laser welding may be considered as a good technique for dental applications in prosthetics, orthodontics, and implantology, even if further studies with different metals and alloys, and also *ex vivo* tests on bovine jaws will be necessary to confirm the results obtained by this work.
